# Severity of inattention symptoms, experiences of being bullied, and school anxiety as mediators in the association between excessive short-form video viewing and school refusal behaviors in adolescents

**DOI:** 10.3389/fpubh.2024.1450935

**Published:** 2024-08-06

**Authors:** Yuru Du, Jianqiang Wang, Ziyan Wang, Jiuying Liu, Shaoxiong Li, Jing Lv, Yuhan Peng, Shining Chang, Miaomiao Li, Huan Liu, Xuan Liu, Xuezhu Yu, Youdong Li

**Affiliations:** ^1^Clinical Mental Health Department, The First Hospital of Hebei Medical University, Hebei, China; ^2^The Key Laboratory of Brain Sciences and Psychology, Hebei, China; ^3^College of Education, Hebei Normal University, Shijiazhuang, China

**Keywords:** excessive short-form video viewing, school refusal behavior, inattention symptoms, being bullied, school anxiety

## Abstract

**Background:**

Recent years have seen an increase in school refusal behavior among adolescents, potentially due to factors like excessive short-form video viewing, bullying, and school anxiety. Limited research has investigated how these factors contribute to school refusal behavior. This study used random forest regression, path analysis, and network analysis to identify key variables and pathways leading to school refusal behavior.

**Methods:**

In this cross-sectional questionnaire-based study, 2,056 (996 male, 1,060 female, mean age: 14.79 ± 1.24 years) middle and senior high school students were asked to complete the School Refusal Behavior Assessment questionnaire to assess school refusal behavior features, the Excessive Short-Form Video Viewing Scale as well as self-reported viewing times during leisure days to assess excessive short-form video viewing, the SNAP-IV Rating Scale to assess the severity of inattention symptoms, and the self-administered questionnaires to assess experiences of being bullied and school anxiety.

**Results:**

The prevalence of school refusal behavior in the surveyed adolescents was found to be 31.9% [95% confidence interval (CI): 29.8–33.9%]. In terms of significance, the severity of inattention symptoms exhibited the greatest predictive power, while excessive short-form video viewing accounted for the most variance. Path analysis revealed that excessive short-form video viewing not only directly affects school refusal behavior features but also does so indirectly through severity of inattention symptoms and school anxiety. Key bridge factors in this pathway include intense fear and anxiety associated with school attendance, manifesting as somatic symptoms and avoidance behaviors.

**Conclusion:**

The findings indicate that not only does excessive short-form video viewing directly influence school refusal behavior features in adolescents, but it also indirectly impacts these features through mechanisms involving severity of inattention symptoms and school anxiety. The bridge factors highlight potential targets for interventions among the SRB features and predictors.

## 1 Introduction

In recent years, there has been a surge in school refusal behavior (SRB) among adolescents, drawing considerable societal attention. SRB refers to adolescents actively refusing to attend school or struggling to stay engaged in classroom learning for the entire day due to psychological or social reasons (excluding physical illness) (1, 2). It includes various features: prolonged absence from school, frequent absenteeism despite the ability to attend, exhibiting disruptive morning behavior such as tantrums or refusal to leave home, and displaying unusual distress at school often leading to requests to avoid attending. A global survey in 2018 revealed that ~17.8% of children/adolescents experience school refusal and related issues (3). In the United States, the prevalence of SRB among adolescents ranged from 5 to 28% in 2001 (4); In Norway, the prevalence of school refusal among students aged 11–15 is ~4% (5); in Japan, there has been a continuous increase in the number of elementary and middle school students exhibiting SRB for eight consecutive years, reaching 244,940 in 2021 (6); while in China, the detection rate of SRB among adolescents was alarmingly high at 22.5% in 2016 (7), with no in-depth scholarly research conducted on this phenomenon post-pandemic. These statistics underscore the global prevalence of SRB, with China facing particularly challenging circumstances. A longitudinal study spanning 15–20 years found that adolescents with SRB have higher rates of psychiatric outpatient referrals, indicating profound implications for their wellbeing (8). Research suggests a close association between adolescent SRB and problematic smartphone use (9). In China, the ownership of online devices among minors has been steadily increasing from 2018 to 2021, with over 60% of adolescents frequently indulging in short-form video content (10). The rise of short-form videos as a prominent form of online entertainment following the pandemic has resulted in widespread adoption among adolescents, contributing to a prevalent trend of excessive short-form video viewing (ESVV). ESVV not only diminishes students' learning satisfaction but also fosters a propensity to evade academic responsibilities (11, 12). Moreover, heightened academic pressures correlate with increased ESVV tendencies (13). Moreover, individuals who engage in ESVV tend to have compromised mental health, often manifesting elevated levels of depression, anxiety, stress, loneliness, and a tendency toward social isolation, especially in terms of severity of inattention symptoms (14). These adverse outcomes associated with ESVV also act as catalysts for the features of SRB, although to date, research detailing the influence of ESVV on the SRB features in adolescents and the underlying mechanisms remains scant. Predictive assessments of these relationships typically rely on theoretical frameworks or empirical evidence. Consequently, this study employs random forest regression and path analysis to investigate the potential influences of ESVV on SRB features and to delineate the potential mechanisms involved.

According to cognitive load theory, human cognition is composed of working memory and long-term memory (15). Viewing short-form videos requires the brain to use working memory to process visual and auditory information continuously. This sustained engagement can overburden the working memory (16), adversely affecting adolescents' ability to allocate attention and maintain focus on other tasks (17). This overload may hinder adolescents from concentrating on their academic tasks, impairing learning functions and potentially leading to SRB features. Furthermore, research indicates that individuals frequently engaged in multitasking with multiple media forms tend to exhibit diminished attentional capacities (18), analogous to the rapid, segmented nature of short-form video content. Consequently, this study hypothesizes that the severity of inattention symptoms (SIS) serves as a mediator in the relationship between ESVV and SRB features.

Previous studies have shown that 20% of students exhibiting SRB attribute this to experiences of being bullied (19). Simultaneously, being bullied can lead to school anxiety (SA), evidenced by anxiety related to school entry and the development of somatic symptoms thereafter, which further manifests as SRB features. Thus, a feasible pathway is that experiences of being bullied (EBB) precipitate SA, subsequently resulting in SRB features. Additionally, research indicates that internet addiction can predict EBB (20, 21), with ESVV as a prominent form, potentially diminishing their face-to-face interactions and support networks, making them targets for bullying, which then leads to SA and SRB features. Further studies suggest that students with significant SIS are more likely to display SRB features when bullied (22), proposing that EBB may mediate the relationship between SIS and SRB features. Therefore, this study hypothesizes that EBB and SA mediate the relationship between ESVV and SRB features, with SIS predicting both EBB and SA.

Considering the significance of these predictors on SRB features, the study utilizes random forest regression to pinpoint unique contributing variables, employs path analysis for hypothesis testing, and conducts network analysis to identify bridging factors within the pathway connections.

## 2 Material and methods

### 2.1 Participants

From June to October 2023, convenience sampling was adopted to survey 2,250 students from five middle and senior high schools in three cities in Hebei Province, China. The inclusion criteria were: (1) age between 12 and 18; (2) currently in school. The exclusion criteria were: (1) students diagnosed with mental disorders in psychiatric outpatient clinics; (2) students with language and communication difficulties. After excluding systematically or incompletely answered questionnaires, 2,056 valid questionnaires were obtained, consisting of 996 males and 1,060 females, with an average age of 14.79 [standard deviation (SD) = 1.24]. Participants were compensated with a stationery set worth 10 RMB upon survey completion. Prior to the survey, Oral informed consent was obtained from the participants, and the study adhered to the Declaration of Helsinki. The study received approval from the ethics committee of the First Hospital of Hebei Medical University (20220933).

### 2.2 Instruments

#### 2.2.1 General information

The general information collected includes gender, age, accommodation status, whether an only-child, and current residence. It also details the school refusal time (SRT) and school refusal due to engagement in more interesting activities (IA).

#### 2.2.2 School Refusal Behavior Questionnaire

The School Refusal Behavior Questionnaire (SRBQ) is utilized to evaluate the severity of SRB features (7), suitable for children aged 5–18. This questionnaire comprises 19 items, scored on a 5-point Likert scale, with total scores ranging from 19 to 95. Higher scores indicate greater severity of SRB features, with scores above 57 denoting the presence of SRB.

#### 2.2.3 Excessive short-form video viewing questionnaire

The metric for assessing ESVV includes the total score from the ESVVQ (23) alongside self-reported short-form viewing times on non-school days (SVT). The questionnaire comprises eight items with a 5-point Likert scoring system, where scores range from 8 to 40—higher scores suggest more severe ESVV. ESVV constitutes a form of internet dependency, akin to gaming disorder yet distinct in that it highlights the frequency and extent of engagement with short-form videos. And short-form videos tend to present opinionated content, whereas games generally do not.

#### 2.2.4 Swanson, Nolan, and Pelham rating scale (SNAP-IV)

The SIS is evaluated using the Inattention subscale from SNAP-IV (24), featuring nine items scored from 0 to 3. The overall score is derived by dividing the total score by the number of items, categorizing scores as normal (0–1), borderline (1.1–1.5), moderate (1.6–1.9), and severe (≥2).

#### 2.2.5 Experiences of being bullied questionnaire

Bullying is categorized into overt bullying, such as verbal or physical abuse, and covert bullying, which includes relational bullying or indirect aggression. According to this classification, the EBBQ is a self-administered questionnaire to evaluate the EBB of adolescents. Covert bullying is assessed by items like “Experiencing verbal abuse, isolation, or instigation of isolation by others online” (EBB-1) and “Being subjected to indirect aggression at school, including sarcasm, verbal abuse, exclusion, or isolation” (EBB-3). Conversely, overt bullying is exemplified by “Being physically assaulted, such as being hit, pinched, or bullied at school” (EBB-2). Each item is rated on a scale from 0 to 4, where a maximum total of 12 indicates the highest severity of EBB.

#### 2.2.6 School anxiety questionnaire

Using selected criteria from the DSM-5 specific to phobias, a school anxiety questionnaire tailored for adolescents school anxiety has been developed (25). This tool comprises three key indicators: “Experiencing anxiety, worry, or tension about going to school” (SA-1), “Manifesting signs of tachycardia, sweating, respiratory difficulties, dizziness, or trembling when at school” (SA-2), and “Engaging in avoidance behaviors such as avoiding or refraining from entering the school premises” (SA-3). Each item is scored from 0 to 4, accumulating a total possible score from 0 to 12, with higher scores reflecting greater degrees of SA (26, 27).

### 2.3 Statistical analysis

We conducted statistical analyses using SASS 27.0 and R 4.3.3.

Firstly, participants were categorized into two groups based on SRBQ scores: those scoring >57 were designated the screening positive SRB group (SP-SRB group), and those scoring ≤ 57 as the screening negative SRB group (SN-SRB group). General information showing significant group differences were treated as covariates. A series of analyses of covariance were utilized to compare differences between the SP-SRB and SN-SRB groups across three factors each of ESVVQ, SVT, SIS, EBB, and SA. Cohen's *d* or partial η2 was computed to gauge effect size, indicating standardized differences or estimates of association between groups.

Secondly, random forest regression analysis was conducted using R 4.4.3 software. General information with significant group differences, ESVV, SIS, factors each of EBB and SA were sequentially entered into the regression model to assess the variance explanation rate, predictive significance, and relative importance of the predictors on SRB features. The “randomForest” package was employed for the regression analysis, and the importance of predictors was estimated using the “rfPermute” package based on 1,000 random permutations. The “percentage of increase of mean square error” [Increase in MS E(%)] was used to measure the relative importance of predictors, with higher values indicating greater importance.

Thirdly, we conducted path analysis using the “lavaan” package in R 4.3.3 to explore the relationships between predictors and SRB features (28). Covariates were defined based on significant differences in general information between groups. In our model, latent variables were shown as ellipses, and observed variables as rectangles. We utilized Bootstrap resampling 5,000 times with a 95% confidence interval to evaluate the significance of the pathways (29). The model's goodness of fit was assessed using several indices: Goodness of Fit Index (GFI), Comparative Fit Index (CFI), Tucker–Lewis Index (TLI), Root Mean Square Error of Approximation (RMSEA), and Standardized Root Mean Square Residual (SRMR), with values exceeding set thresholds (GFI > 0.90, CFI > 0.90, TLI > 0.90, RMSEA <0.08, SRMR <0.05) indicating a satisfactory fit (30).

Lastly, we performed a network analysis using the “qgraph” package in R 4.4.3, incorporating ESVV, SRB features, and significant mediating variables as nodes. We calculated the bridge expected influence using the “networktools” package, where higher values indicated an increased risk of the variable affecting others. The most influential factors (top 10%) were considered key bridge factors. Stability of the network model was tested through 1,000 Bootstrap replications with each variable, and the model's robustness was evaluated using Convergent Stability (CS), where CS values > 0.5 denote strong stability. In our network diagrams, positive correlations were illustrated with green lines and negative correlations with red lines, with thicker lines indicating stronger relationships.

## 3 Results

### 3.1 Group differences in general information

Based on the SRBQ criteria for SRB evaluation, it was found that 655 adolescents exhibited SRB (SP-SRB group) compared to 1,401 who did not (SN-SRB group), yielding an SRB detection rate of 31.9% [95% confidence interval (CI): 29.8–33.9%]. No significant gender differences were observed between the SP-SRB and SN-SRB groups. Adolescents in the SP-SRB group tended to be older and were more frequently non-only children, lived in rural areas, and had longer periods of SRT. Additionally, those who refused school to engage in more interesting activities were more likely to display SRB (all *P* < 0.05, Table 1).

**Table 1:** Differences in general variables between the SN-SRB group and SP-SRB group.

	**SN-SRB group (*n* = 1,401)**	**SP-SRB group (*n* = 655)**	**Test**	***P***	
Gender (*n*)			χ^2^ = 2.385	0.123	
Male	695	301			
Female	706	354			
Age (years), mean (SD)	14.61 (1.28)	15.16 (1.06)	*t* = −10.122	^***^	0.448^a^
Whether accommodated			χ^2^ = 115.934	^***^	
Yes	899	571			
No	502	84			
Only-child			χ^2^ = 22.191	^***^	
Yes	222	54			
No	1,179	601			
Current residence			χ^2^ = 51.518	^***^	
Urban	570	160			
Rural	831	495			
School refusal time (SRT)			χ^2^ = 21.590	^***^	
0 day	1,231	540			
<7 days	149	93			
7–14 days	17	11			
14–21 days	1	0			
21–28 days	3	11			
Interesting activities (IA)			χ^2^ = 116.948	^***^	
Yes	113	168			
No	1,288	487			

^a^|Cohen's *d*|, absolute value of Cohen's *d*; small effect size: 0.20 <|Cohen's *d*| <0.50; middle effect size: 0.50 ≤ |Cohen's *d*| <0.80; large effect size: |Cohen's *d*| ≥ 0.80.

^***^P < 0.001.

### 3.2 Group differences in ESVV, SIS, factors of EBB and SA

**Figure 1:**
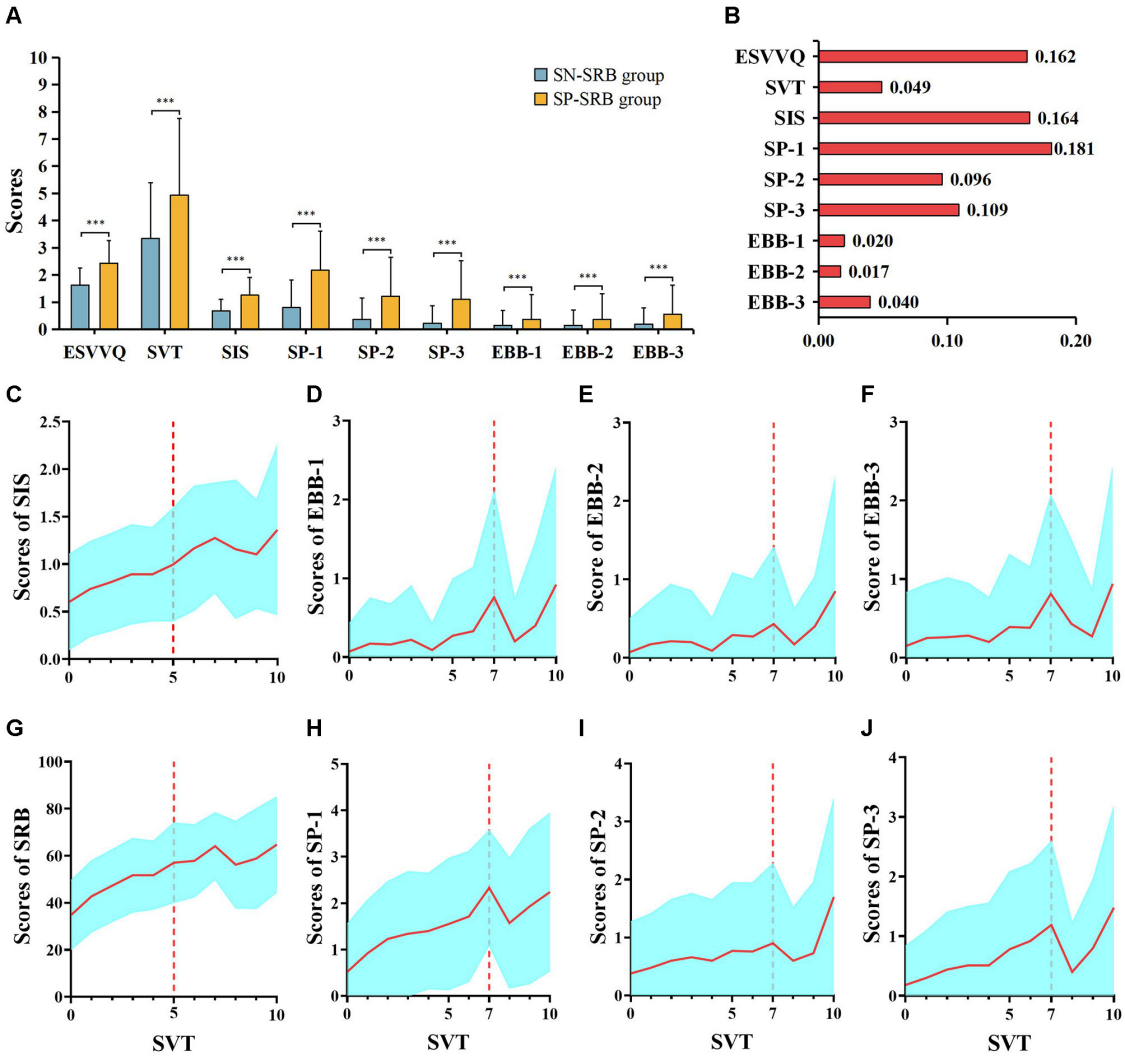
Group differences in ESVV, SIS, factors of EBB or SA. **(A)** SP-SRB and SN-SRB groups differences in these predictors. **(B)** Effect sizes of ANCOVA analyses in predictors between SP-SRB and SN-SRB groups. The Partial η2 was calculated to evaluate the effect size. Small effect size: 0.01 <partial η2 <0.06; middle effect size: 0.06 ≤ partial η2 <0.14; large effect size: partial η2 ≥ 0.14. **(C–J)** Relationship between SIS, factors of SA, SRB features, factors of EBB and SVT. ***P < 0.001.

The SP-SRB group showed significantly higher severity levels related to ESVVQ, SVT, and SIS, as well as in the factors associated with EBB and SA, than the SN-SRB group (Figure 1A). Notably, the effect sizes for ESVVQ, SIS, and SA-1 were relatively large (Figure 1B).

The SVT metric, which primarily measures screen time, indicates that exceeding 5 h of SVT progressively leads to symptoms of inattention (Figure 1C) and to increasingly engage in SRB (Figure 1G). Furthermore, when SVT surpasses 7 h, peaks in EBB (Figures 1D–F) and SA (Figures 1H–J) are first observed.

### 3.3 Random forest regression analysis

**Figure 2:**
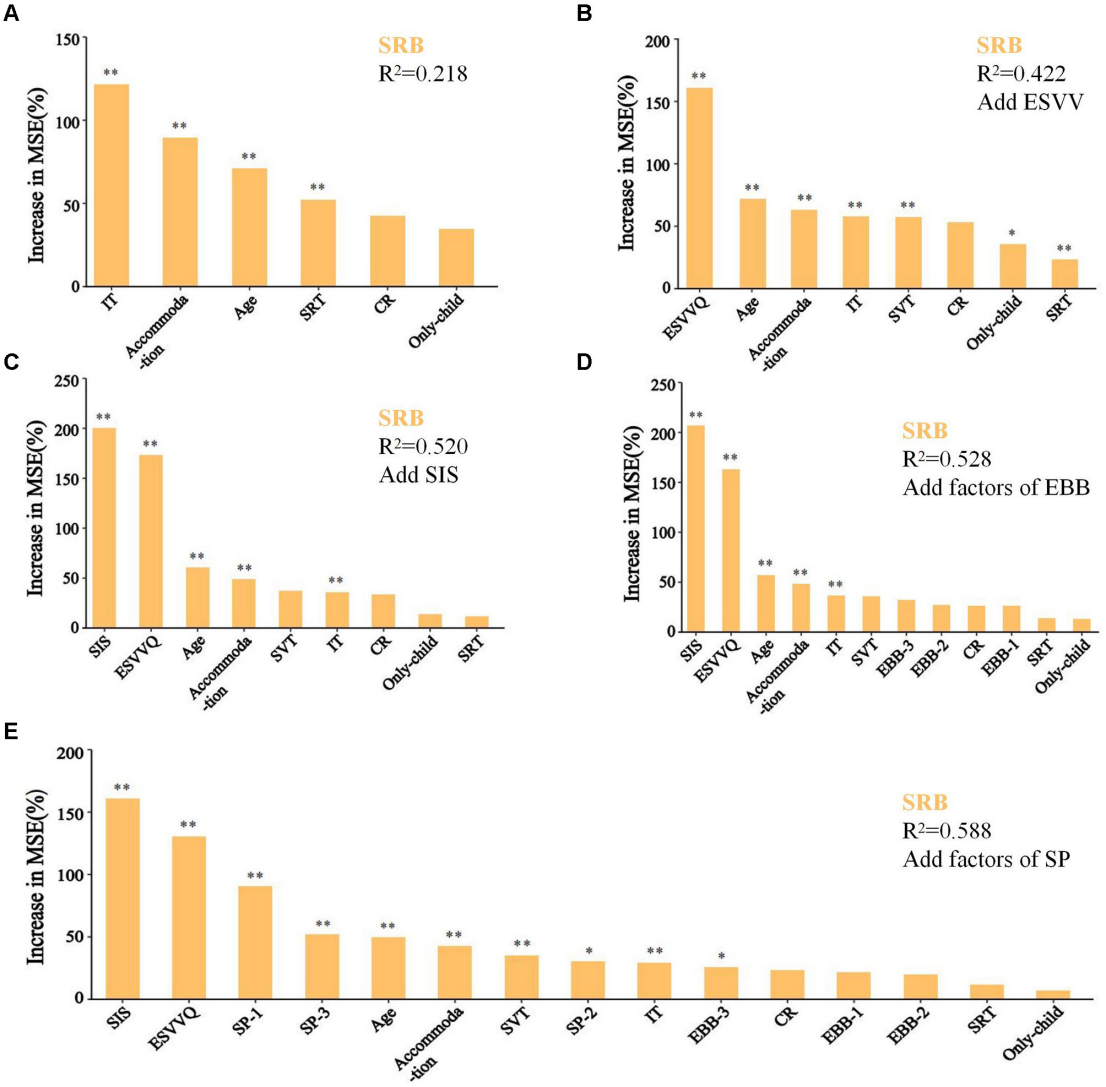
Random forest regression analysis. **(A–E)** General information, ESVV, SIS, and three specific factors from EBB and SA were gradually incorporated into the prediction of SRB features. Percentage increases in the MSE (mean squared error) of variables were used to estimate the importance of these predictors, and higher MSE% values imply more important predictors. *P < 0.05, **P < 0.01.

Using a random forest regression analysis, significant variables from general information, along with ESVV, SIS, and three specific factors each from EBB and SA, were sequentially analyzed to determine their impact on the SRB features (Figure 2). General information explained 21.8% of the variance in SRB features, with variables such as IA, accommodation status, age, and SRT emerging as significant predictors (all *P* < 0.01) (Figure 2A). Further inclusion of the ESVV, SIS, EBB, and SA factors saw the explained variance of SRB features increasing to 0.422, 0.520, 0.528, and 0.588, respectively, representing increases of 0.204, 0.098, 0.008, and 0.060. ESVV made the largest contribution to explaining the variance in SRB features (Figures 2B–E). As illustrated in Figure 2E, variables such as SIS, ESVVQ, SVT, three specific factors of SA, EBB-3, age, whether accommodation, and IA were all significant predictors of SRB features (all *P* < 0.05). However, EBB-1 and EBB-2 were not significant predictors of SRB features. Among the predictors, SIS was the most important, followed by ESVVQ (Figure 2E).

### 3.4 Path analysis: the hypothesis mediation model of SRB

**Figure 3:**
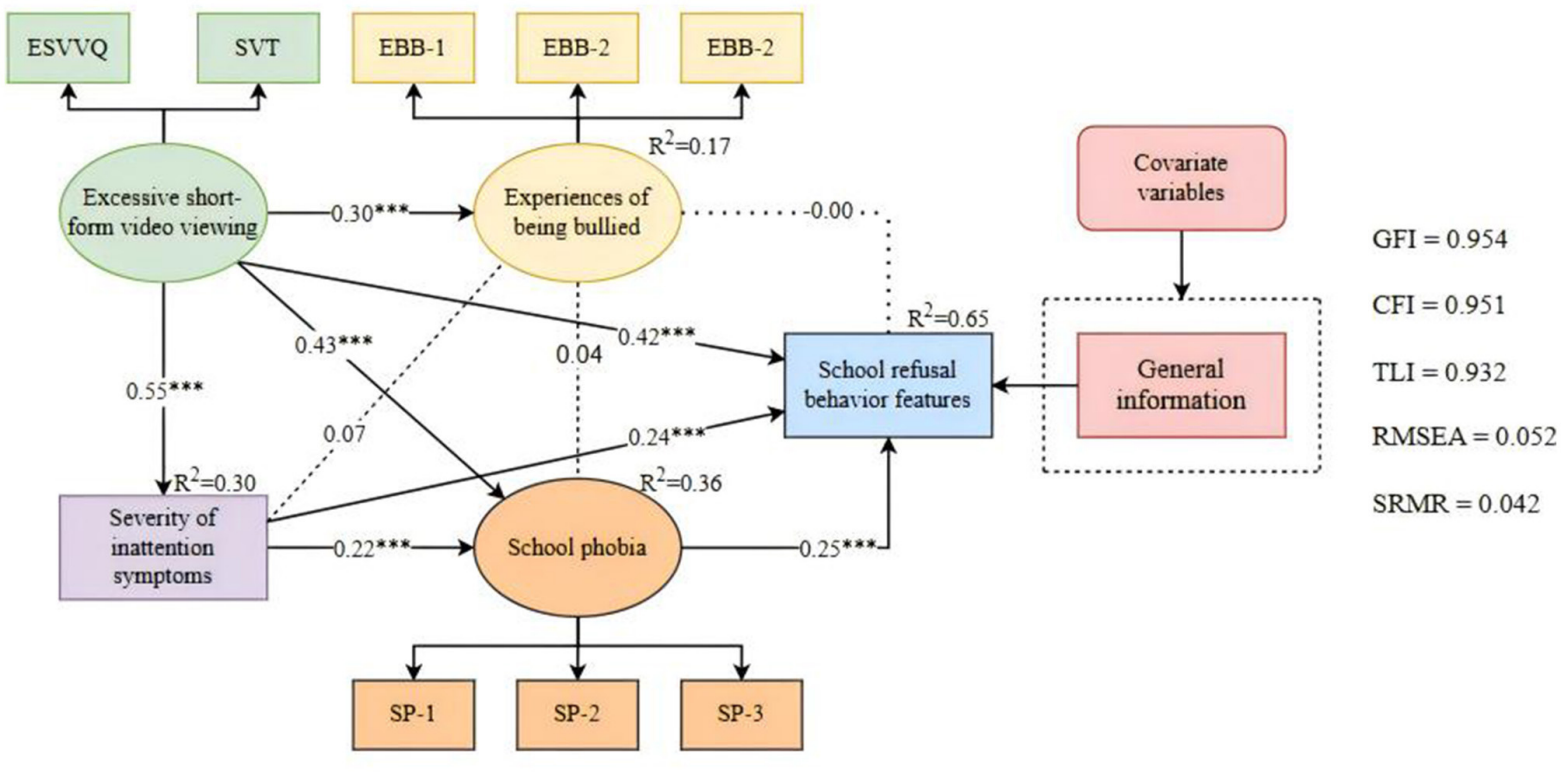
The hypothesis mediation model. The hypothesis model was evaluated by maximum likelihood estimation with 5,000 Bias-corrected bootstrapped. ***P < 0.001.

The hypothesized model in this study demonstrates a strong fit, with a multiple correlation coefficient of 0.65 for SRB features, indicating a good predictive power of the model. Figure 3 illustrates that certain variables, namely ESVV, SIS, and SA, directly influence SRB features, while EBB does not. However, given the lack of significant relationships between SIS and EBB, and between EBB and SA, ESVV indirectly impacts SRB features via three pathways (with a 95% confidence interval excluding 0, as shown in Figure 3 and Table 2). The most influential path is ESVV exacerbating inattention symptoms, thereby impacting SRB features (Estimate = 0.385), followed by ESVV leading to SA, subsequently affecting SRB features (Estimate = 0.313).

**Table 2:** Standardized indirect effects and 95% confidence intervals.

**Path**	**Estimate**	**95%CI**	***P***
**Hypothesis mediation model**			
ESVV → EBB → SRB features	**–**0.003	**–**0.045, 0.030	0.872
ESVV → SA → SRB features	0.313	0.233, 0.404	^***^
ESVV → SIS → SRB features	0.385	0.313, 0.463	^***^
ESVV → SIS → EBB → SRB features	0.000	**–**0.007, 0.005	0.885
ESVV → SIS → SA → SRB features	0.089	0.054, 0.126	^***^
ESVV → EBB → SA → SRB features	0.010	**–**0.005, 0.026	0.224
ESVV → SIS → EBB → SA → SRB features	0.001	**–**0.001, 0.004	0.338
Total indirect effects	0.795	0.684, 0.912	^***^

^***^P < 0.001.

### 3.5 Network analysis

**Figure 4:**
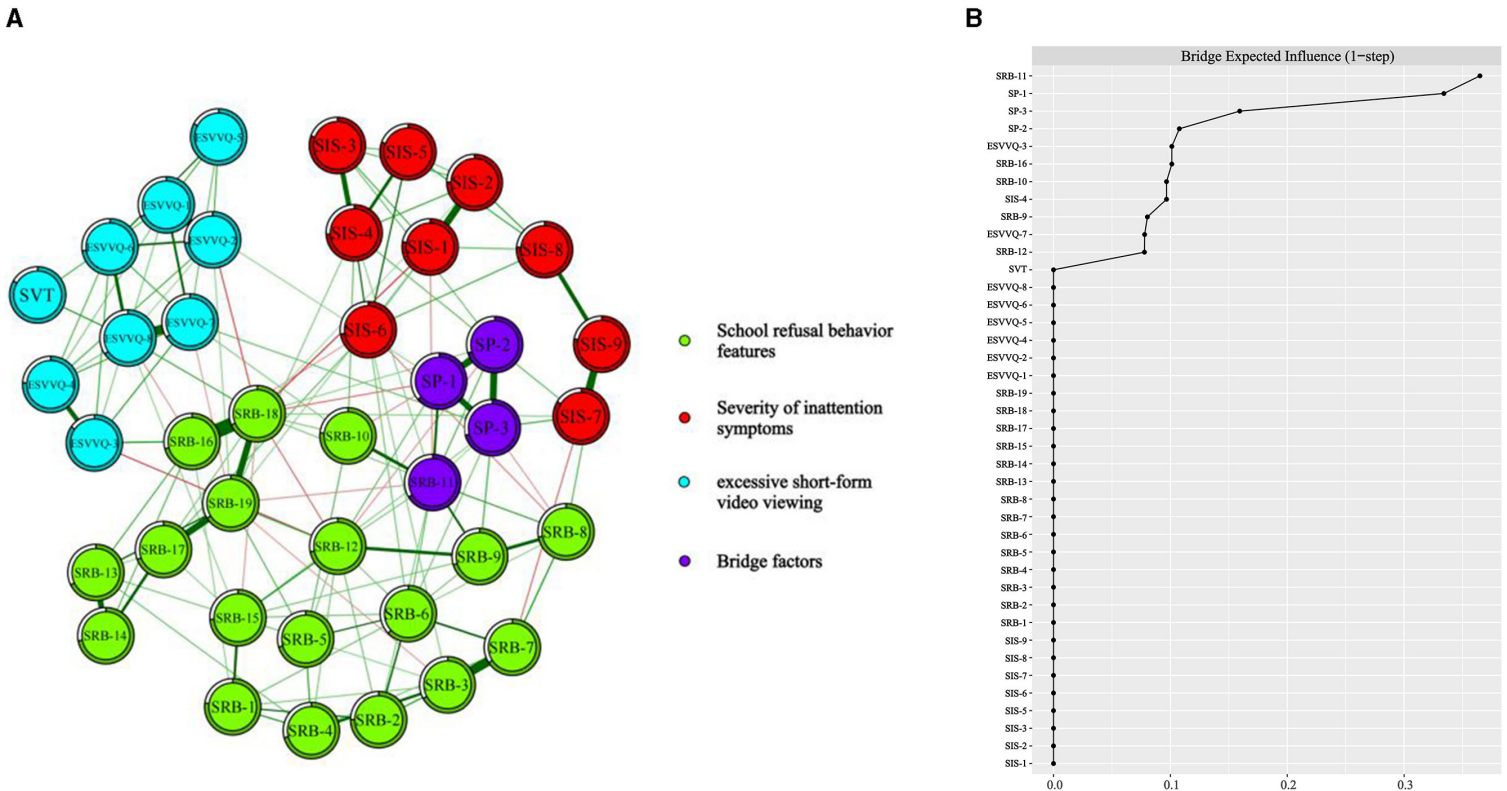
Network analysis of ESVV, SIS, SA, and SRB features. **(A)** Network structure; **(B)** The bridge expected influence values of each node, with nodes positioned increasingly to the right on the horizontal axis indicating higher values of bridge expected influence.

Network analysis was performed with ESVV, SRB features, and mediating variables (SIS and the factors of SA) as nodes (Figure 4A). The analysis revealed that the factors with the highest bridge expected influence scores are SRB-11 (Attending school induces anxiety and fear, bridge expected influence = 0.36), SA-1 (Bridge expected influence = 0.33), SA-3 (Bridge expected influence = 0.16), and SA-2 (Bridge expected influence = 0.11). Reducing the impact of these key bridge factors can decrease variable interactions within this network structure, suggesting that targeted interventions could be effective. The correlation stability coefficient was 0.67, indicating excellent stability for the nodes in the network. The bridge expected influence values for other factors are shown in Appendix 1.

## 4 Discussion

This study applied machine learning, path analysis, and network analysis to first examine the impact of ESVV, SIS, EBB, and SA on SRB features. It also evaluated potential paths of impact and the bridge factors that connect these paths. The random forest regression identified ESVV as the most significant predictor of variability in adolescent SRB features. Path analysis demonstrated that all predictors, except for EBB, could predict SRB features, with ESVV influencing SRB features through three indirect pathways. Subsequent network analysis identified that SRB-11 and the three factors of SA serve as key bridge factors in the network structure. In summary, the findings suggest that ESVV in adolescents may exacerbate inattention symptoms and increase SA, contributing to SRB features, with SRB-11 and the three SA factors acting as pivotal connectors in this pathway.

ESVV directly affects features of SRB. Additionally, displaying SRB features is noted when the SVT reaches 5 h. Watching short-form videos tailored to adolescents' preferences leads to increased dopamine secretion (31, 32). Long-term exposure to these videos accustoms the brain to high dopamine levels, ultimately leading to dopamine tolerance (33, 34). Dopamine, often referred to as the “pleasure molecule,” also embodies desire, driving individuals toward engaging activities (35). Upon returning to school, traditional activities like reading, attending classes, and sports no longer provide the same level of pleasure previously experienced, thus diminishing their appeal and leading to the manifestation of SRB features. This suggests that the pathway through which ESVV influences SRB features in adolescents may be linked to dopamine tolerance.

ESVV not only directly impacts SRB features but also exerts indirect influence through three pathways. Among these pathways, SIS emerges as the primary mediator in triggering SRB features induced by ESVV. The SP-SRB group, in contrast to the SN-SRB group, reported prolonged and excessive short-form video viewing, with attention symptoms manifesting when SVT reaches 5 h. Moreover, the pathway effect size of SRB features triggered by SIS is significant. These findings suggest that SIS plays a mediating role in ESVV's impact on adolescent SRB features. The continuous influx of short-form videos into the brain activates the reward pathway, releasing dopamine, while simultaneously inhibiting the thalamus responsible for attention allocation and inhibitory control, thereby diminishing individuals' self-control over attention (32). As attention naturally gravitates toward stimuli that activate dopamine neurons, such as short-form videos, shifting attention to campus life disrupts this dopamine response, akin to associations between campus life and punishment (36, 37). At the same time, according to the classical attention bias theory, when adolescents engage in prolonged viewing of short-form videos on weekends, they are likely to redirect their attention toward stimuli that offer instant pleasure or satisfaction upon their return to school. In contrast, learning tasks that necessitate sustained attention and can be perceived as somewhat monotonous are unlikely to furnish them with immediate gratification. Consequently, when ESVV leads to dopamine tolerance, attention struggles to remain engaged in “low dopamine activities” like campus life, resulting in attention symptoms. This aligns with observations in Nature that multitasking with various media tasks often leads to attention deficits (18).

The second key mediator of the influence of ESVV on SRB features is SA. The SP-SRB group experiences more severe SA than the SN-SRB group, with SA peaking when SVT reaches 7 h. ESVV is considered a form of addictive behavior (38), and adolescents affected by ESVV may exhibit a series of physical symptoms related to SA—such as accelerated heartbeat, sweating, breathing difficulties, dizziness, or trembling—likely due to withdrawal symptoms from not accessing short-form videos (39). Therefore, it is hypothesized that SA plays a significant role in mediating the impact of ESVV on SRB features through mechanisms related to dopamine withdrawal.

EBB does not serve as a mediating factor in the relationship between ESVV and SRB features. Covariance analysis indicates that the SP-SRB group's EBB scores are significantly higher than those of the SN-SRB group. However, only covert bullying, specifically EBB-3, predicts SRB features, as shown by random forest regression. Path analysis further demonstrates that EBB cannot predict SA and SRB features, nor can SIS predict EBB, and EBB does not play a mediating role between ESVV and SRB features. Two potential explanations emerge: (1) In Asian cultural contexts, victims may be reluctant to self-report EBB, particularly when bullies are present within the school environment, due to the stigma associated with being bullied (40). (2) Students who have experienced overt bullying might avoid reporting their experiences due to fears of further bullying or retaliation (41). Additionally, path analysis suggests that ESVV predicts adolescent EBB, as excessive video watching during free days reduces time for offline peer interactions, impeding the development of stable social support networks (42). Research indicates that ESVV triggers negative emotional states in adolescents, such as depression and decreased motivation, which may lead to social isolation as they are perceived as “outsiders” by their peers (38, 43).

Network analysis has identified that SRB-11 and three factors of SA serve as central bridge factors within the network, reflecting negative schooling experiences manifested through anxiety, physical symptoms, and avoidance behaviors. Tekin and Aydin's meta-analysis underscores a direct, substantial linkage between SRB and various forms of anxiety, including state, trait, social, school, and separation anxieties (44). The presence of somatic symptoms, frequently accompanying anxiety, directly contributes to the emergence of SRB (45, 46). Reducing anxiety, avoidance, and somatic symptoms at school could therefore mitigate the adverse effects of ESVV and lower the occurrence of SRB features.

This study establishes that ESVV directly impacts adolescents' SIS, EBB, SA, and SRB features and indirectly affects SRB through SIS and SA. The detrimental impacts are conjectured to relate to dopamine tolerance, suggesting a progressive approach to dopamine tolerance detoxification could facilitate the reintegration of school refusal adolescents into academic settings. This could involve structuring activities to balance low and high dopamine activities initially at a 4:1 ratio, gradually adjusting this balance to moderate dopamine output and prevent intense withdrawal symptoms.

Nevertheless, the study has limitations, notably its cross-sectional nature, which precludes establishing causality between ESVV, SIS, SA, and SRB features. Additionally, the proposed role of dopamine in these processes remains speculative, necessitating further empirical validation.

## Data availability statement

The raw data supporting the conclusions of this article will be made available by the authors, without undue reservation.

## Ethics statement

The studies involving humans were approved by the Ethics Committee of the First Hospital of Hebei Medical University. The studies were conducted in accordance with the local legislation and institutional requirements. The Ethics Committee/Institutional Review Board waived the requirement of written informed consent for participation from the participants or the participants' legal guardians/next of kin because Biological samples were not involved in this study.

## Author contributions

YD: Investigation, Formal analysis, Data curation, Writing – review & editing, Writing – original draft, Methodology, Conceptualization. JW: Writing – review & editing, Writing – original draft, Methodology, Investigation, Formal analysis, Data curation, Conceptualization. ZW: Writing – review & editing, Writing – original draft, Methodology, Investigation, Formal analysis. JLi: Software, Writing – review & editing, Methodology, Investigation. SL: Validation, Supervision, Writing – review & editing, Software. JLv: Writing – review & editing, Validation, Supervision, Software. YP: Writing – review & editing, Validation, Supervision, Software. SC: Visualization, Project administration, Writing – review & editing, Supervision. ML: Writing – review & editing, Visualization, Supervision, Project administration. HL: Software, Investigation, Writing – review & editing. XL: Writing – review & editing, Software, Investigation. XY: Resources, Methodology, Funding acquisition, Conceptualization, Writing – review & editing, Software. YL: Writing – original draft, Writing – review & editing, Software, Resources, Methodology, Funding acquisition, Conceptualization.
