# Supplementary material for: Severity of inattention symptoms, experiences of being bullied, and school anxiety as mediators in the association between excessive short-form video viewing and school refusal behaviors in adolescents
Source: Front Public Health. 2024 Aug 6;12:1450935. doi: 10.3389/fpubh.2024.1450935 (PMC11337196; doi:10.3389/fpubh.2024.1450935)
Supplement: Supplementary file 1 [file Table_1.DOCX]

**Supplement Table 1**

| Item abbreviation | Item content | Bridge expected influence |
| --- | --- | --- |
| SRB-1  SRB-2  SRB-3  SRB-4  SRB-5  SRB-6  SRB-7  SRB-8  SRB-9  SRB-10  SRB-11  SRB-12  SRB-13  SRB-14  SRB-15  SRB-16  SRB-17  SRB-18  SRB-19  SP-1  SP-2  SP-3  ESVVQ-1  ESVVQ-2  ESVVQ-3  ESVVQ-4  ESVVQ-5  ESVVQ-6  ESVVQ-7  ESVVQ-8  SVT  SIS-1  SIS-2  SIS-3  SIS-4  SIS-5  SIS-6  SIS-7  SIS-8  SIS-9 | Feeling tired and lacking energy even after waking up in the morning.  Time seems to drag on while at school.  Remembering what was taught in class is challenging.  Feeling drowsy during lectures.  Maintaining focus in class or while doing homework is less effective compared to playing on the computer or mobile phone.  Attending school is an exhausting activity.  Grasping class material is difficult.  Misfortunes seem to occur frequently at school.  Thinking about school over the weekend brings a bad mood.  Completing homework requires supervision.  Attending school induces anxiety and fear.  Eager to leave school immediately after classes end.  Engaging in non-academic activities when disinterested in class.  Skipping difficult problems in homework.  Waking up in the morning for school is challenging.  It is enjoyable to defy the teacher.  Optional exercises, apart from mandatory homework, can be left undone.  Disrupting class can be entertaining.  Homework may be neglected if the teacher does not check it.  Experiencing anxiety, worry, or tension about going to school.  Manifesting signs of tachycardia, sweating, respiratory difficulties, dizziness, or trembling when at school  Engaging in avoidance behaviors such as steering clear of or refraining from entering the school premises  When I am not utilizing the short-form video application, it often preoccupies my thoughts.  I engage with the short-form video application frequently without a specific rationale.  I have experienced conflicts with others due to my excessive use of the short-form video application.  If I desire to use the short-form video application, I immediately cease my current activities.  Engaging with the short-form video application enhances my sense of connectedness with others.  I have lost track of the frequency with which I use the short-form video application.  The notion of being unable to access the short-form video application causes significant psychological distress.  I am unable to mitigate my usage of the short-form video application.  Short-form videos time  Demonstrates an inability to concentrate on detail-oriented tasks, frequently making careless errors in schoolwork and other activities.  Exhibits difficulty maintaining attention in task-oriented or recreational activities.  Often does not seem to register what others are saying.  Incapable of adhering to instructions, consistently failing to complete school assignments or household tasks.  Struggles with organizing tasks and planning activities.  Tends to avoid, shows reluctance, or struggles significantly with tasks that require sustained mental effort, such as school assignments or homework.  Regularly misplaces items essential for tasks and activities, such as school assignments, pencils, books, tools, or toys.  Is readily distracted by irrelevant external stimuli.  Frequently forgets tasks required in daily routines. | 0  0  0  0  0  0  0  0  0.08  0.10  0.36  0.08  0  0  0  0.10  0  0  0  0.33  0.11  0.16  0  0  0.10  0  0  0  0.08  0  0  0  0  0  0.10  0  0  0  0  0 |
